# Non-contrast assessment of microvascular integrity using arterial spin labeled cardiovascular magnetic resonance in a porcine model of acute myocardial infarction

**DOI:** 10.1186/s12968-018-0468-5

**Published:** 2018-07-02

**Authors:** Hung P. Do, Venkat Ramanan, Xiuling Qi, Jennifer Barry, Graham A. Wright, Nilesh R. Ghugre, Krishna S. Nayak

**Affiliations:** 1 Department of Physics and Astronomy, University of Southern California, 3740 McClintock Ave, EEB 400, Los Angeles, California 90089-2564 USA; 2 Physical Sciences Platform, Sunnybrook Research Institute, Toronto, ON Canada; 3 Department of Medical Biophysics, University of Toronto, Toronto, ON Canada; 4 Schulich Heart Research Program, Sunnybrook Health Sciences Centre, Toronto, ON Canada; 5 Ming Hsieh Department of Electrical Engineering, University of Southern California, Los Angeles, CA USA

**Keywords:** Myocardial blood flow, Arterial spin labeling, Acute myocardial infarction, Microvascular obstruction, Microvascular integrity, Non-contrast myocardial perfusion imaging, Vasodilator response

## Abstract

**Background:**

Following acute myocardial infarction (AMI), microvascular integrity and function may be compromised as a result of microvascular obstruction (MVO) and vasodilator dysfunction. It has been observed that both infarcted and remote myocardial territories may exhibit impaired myocardial blood flow (MBF) patterns associated with an abnormal vasodilator response. Arterial spin labeled (ASL) CMR is a novel non-contrast technique that can quantitatively measure MBF. This study investigates the feasibility of ASL-CMR to assess MVO and vasodilator response in swine.

**Methods:**

Thirty-one swine were included in this study. Resting ASL-CMR was performed on 24 healthy swine (baseline group). A subset of 13 swine from the baseline group underwent stress ASL-CMR to assess vasodilator response. Fifteen swine were subjected to a 90-min left anterior descending (LAD) coronary artery occlusion followed by reperfusion. Resting ASL-CMR was performed post-AMI at 1–2 days (*N* = 9, of which 6 were from the baseline group), 1–2 weeks (*N* = 8, of which 4 were from the day 1–2 group), and 4 weeks (*N* = 4, of which 2 were from the week 1–2 group). Resting first-pass CMR and late gadolinium enhancement (LGE) were performed post-AMI for reference.

**Results:**

At rest, regional MBF and physiological noise measured from ASL-CMR were 1.08 ± 0.62 and 0.15 ± 0.10 ml/g/min, respectively. Regional MBF increased to 1.47 ± 0.62 ml/g/min with dipyridamole vasodilation (*P* < 0.001). Significant reduction in MBF was found in the infarcted region 1–2 days, 1–2 weeks, and 4 weeks post-AMI compared to baseline (*P* < 0.03). This was consistent with perfusion deficit seen on first-pass CMR and with MVO seen on LGE. There were no significant differences between measured MBF in the remote regions pre and post-AMI (*P* > 0.60).

**Conclusions:**

ASL-CMR can assess vasodilator response in healthy swine and detect significant reduction in regional MBF at rest following AMI. ASL-CMR is an alternative to gadolinium-based techniques for assessment of MVO and microvascular integrity within infarcted, as well as salvageable and remote myocardium. This has the potential to provide early indications of adverse remodeling processes post-ischemia.

## Background

Microvascular obstruction (MVO) is a common complication after acute myocardial infarction (AMI) [1]. MVO is described as a “no-reflow” phenomenon [2, 3], in which myocardial blood perfusion is impaired at the capillary level even after reperfusion. Recent studies have established that MVO is independently associated with adverse ventricular remodeling and patient prognosis. Hence MVO detection and monitoring are crucial, especially in high-risk patients [4–6]. Additionally, microvascular function after an AMI is often compromised where vasodilator response is impaired not only in the infarcted but also in the remote myocardial territories [7].

Since MVO is defined as a “no-reflow” phenomenon, quantitative measurement of myocardial blood flow (MBF) would be a direct measure of MVO and its severity. Several techniques such as microspheres, computed tomography, positron emission tomography (PET), single photon emission computed tomography (SPECT), and gadolinium-based first-pass cardiovascular magnetic resonance (CMR) have been used for quantitative assessment of myocardial perfusion [8]. Microspheres is the gold standard for assessment of tissue perfusion but it is invasive requiring organ extraction and hence not directly applicable for clinical use [9]; however, it is highly instrumental for validation studies. The other imaging modalities are able to measure MBF noninvasively, however, they have limitations of either involving ionizing radiation and/or require the use of exogenous contrast agents.

Arterial spin labeling CMR (ASL-CMR) [10] is a non-contrast CMR technique that can quantitatively assess myocardial blood flow (MBF) in small animals [11–15], large animals [16, 17] and humans [18–20]. ASL-CMR is capable of detecting clinically relevant increases in MBF with vasodilation and has shown potential for diagnosing coronary artery disease in patients [21, 22]. ASL-CMR does not involve ionizing radiation or require the use of exogenous contrast agents therefore it can be performed repeatedly or even continuously [23, 24]. In this work, we aimed to investigate the feasibility of ASL-CMR to assess MVO and vasodilator response in swine.

## Methods

### Animal protocol

Our study utilized female Yorkshire swine (*N* = 31, 20–25 kg) obtained from Caughell Farms (Ontario, Canada) and the animal protocol was approved by the Animal Care Committee of Sunnybrook Research Institute. Prior to all interventional procedures and CMR imaging, swine were intubated and sedated using an anesthetic cocktail of atropine (0.05 mg/kg) and ketamine (30 mg/kg). Respiration was controlled (20–25 breaths/min) using a mechanical ventilator and isoflurane (1–5%) was administered to maintain the anesthetic plane throughout an experiment. Fifteen swine underwent the AMI procedure, in which the left anterior descending coronary artery (LAD) was completely occluded for 90 min just beyond the second diagonal branch using a percutaneous balloon dilation catheter (Sprinter Legend Balloon Catheter, Medtronic, Minneapolis, Minnesota, USA). After 90 min, the balloon was released, and the vessel was allowed to reperfuse. The interventional procedures were performed under X-ray fluoroscopy (Philips Veradius, Philips Healthcare, Best, the Netherlands) to guide balloon placement and inflation and verify reperfusion. Swine were allowed to recover for subsequent CMR imaging.

### CMR imaging

All experiments were performed on a 3 T scanner (MR750, General Electric Healthcare, Waukesha, Wisconsin, USA) with an 8-channel cardiac receiver coil. The general scan protocol and imaging times are listed in Table 1. CMR imaging was performed at baseline (healthy state), 1–2 days, 1–2 weeks, and 4 weeks post-AMI.

**Table 1:** Cardiovascular magnetic resonance (CMR) protocol

Scan time	CMR Protocol
3 min	Localization
10 min	cine (12–14 short-axis, 2–5 long-axis)
3 min	ASL-CMR (Rest)
3 min	ASL-CMR (Stress)
1 min	First-pass CMR
5 min	LGE CMR (8 min post Gad injection)

*ASL* arterial spin labeling, *CMR* cardiovascular magnetic resonance, *LGE* late gadolinium enhancement

Cardiac function was assessed using a cine balanced steady-state-free-precession (bSSFP) sequence with the following parameters: 12–14 short-axis slices, 3–5 long-axis slices, TR/TE = 4.0/1.7 ms, flip angle = 45°, field-of-view = 24 × 21.6 cm^2^, acquisition matrix = 224 × 192, bandwidth = 125 kHz, 8 views-per-segment and 20 cardiac phases.

ASL-CMR was performed at mid-ventricular short axis slices identified from 3-chamber and 4-chamber cine scout images. Each ASL-CMR scan was composed of seven breath-holds and took approximately 3 min. An image without labeling pulse and a noise image were acquired in the first 3-s breathhold. Six pairs of control and labeled images were acquired with 12-s breathholds. Flow-sensitive alternating inversion recovery (FAIR) [25, 26] was implemented for this study, in which a nonselective and a 30 mm slice-selective hyperbolic secant adiabatic inversion pulses were applied 2 heartbeats (i.e. post-labeling-delay is 2 RR) prior to a bSSFP image acquisition to obtain labeled and control images, respectively. The FAIR labeled and control pulses and the center of image acquisition were triggered to mid-diastole. Trigger timing was defined based on cine scout images. Heart rate was recorded in all ASL-CMR scans. Imaging parameters were: bSSFP, TR/TE = 3.2/1.5 ms, flip angle = 50°, slice thickness = 10 mm, field-of-view = 18–24 cm^2^, acquisition matrix = 128 × 128, bandwidth = 62.5 kHz, SENSE parallel imaging rate 2 [27]. A 19-TR Kaiser–Bessel weighted [28] ramp-up and ramp-down were used to optimally minimize transient artifact and preserve longitudinal magnetization after image acquisition, respectively. A 12 ms fat-saturation pulse was used prior to the ramp-up pulses.

First-pass CMR was performed using a multiphase fast gradient-echo sequence to capture the first passage of the contrast agent (8–12 mL of Gadolinium-DTPA 0.2 mmol/kg; Magnevist, Bayer Pharmaceuticals, Berlin, Germany). The sequence parameters of first-pass CMR were TR/TE = 2.8/1.3 ms, slice thickness = 7 mm, slice spacing = 3 mm, flip angle = 20°, acquisition matrix = 128 × 128. LGE imaging was performed 8 min after contrast injection using a T1-weighted inversion recovery gradient-echo sequence with the following parameters: TR/TE = 4.1/1.9 ms, flip angle = 15°, acquisition matrix = 224 × 192, 2RR intervals; the inversion time was adjusted to null the signal from normal myocardium (TI = 280–320 ms).

### Data collection

**Fig. 1:**
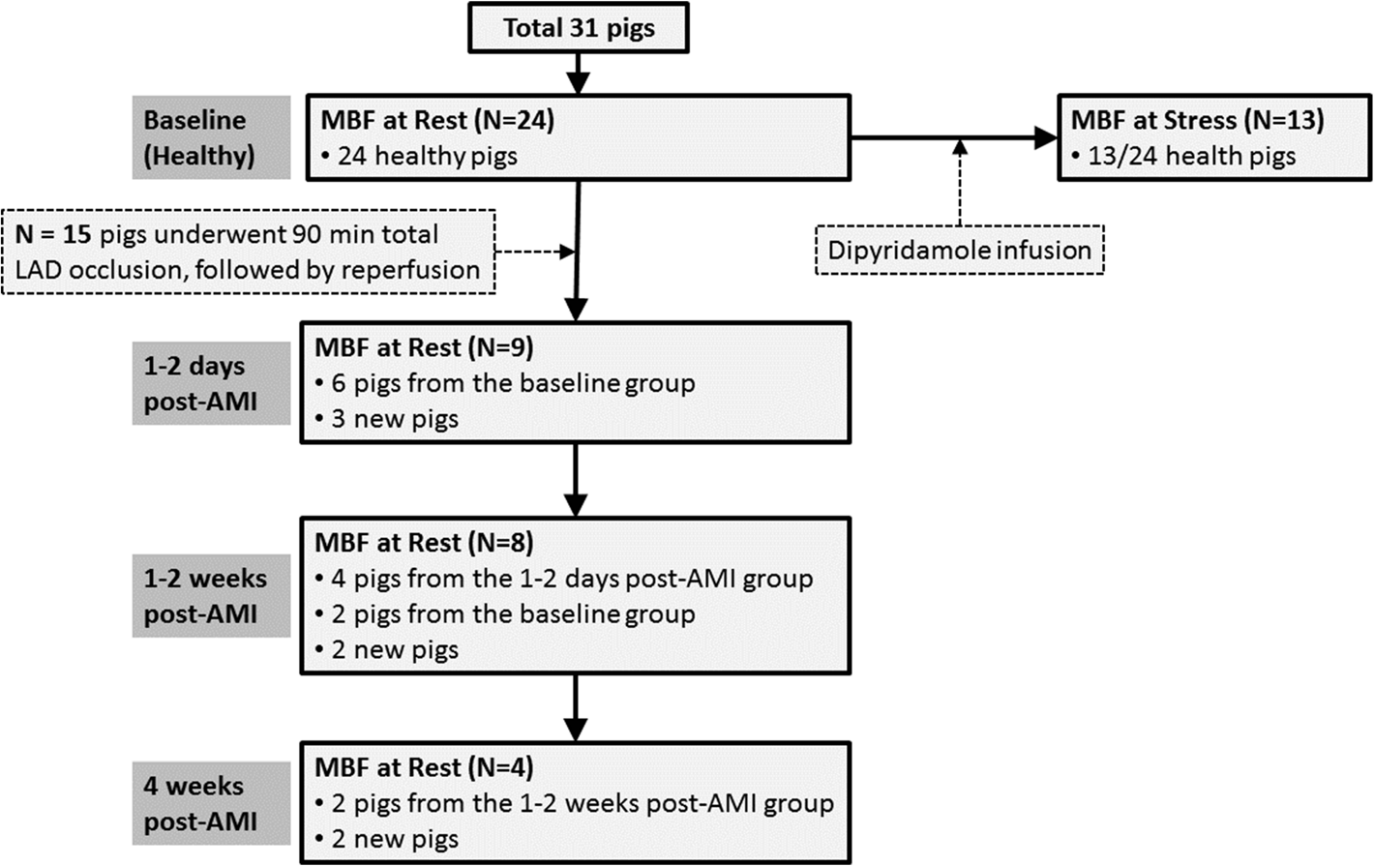
Data collection process showing different animal groups underwent arterial spin labeling cardiovascular magnetic resonance (ASL-CMR) at different conditions and times. Cross-sectional analysis was used when comparing regional myocardial blood flow (MBF) measured post-acute myocardial infarction (AMI) to that measured at baseline since every animal was not imaged at all time points

Thirty-one swine were included in this study. Data collection process and animal utilization are outlined in Fig. 1. At baseline (healthy state), 24 swine underwent ASL-CMR at rest. In each swine, 1–3 short axis slices were acquired resulting in a total of 41 short axis slices. To assess vasodilator response, 13 out of 24 healthy swine subsequently underwent stress ASL-CMR. Stress ASL-CMR was performed 4 min after intravenous injection of the pharmacologic vasodilator dipyridamole (Pharmaceutical Partners of Canada, Toronto, Ontario, Canada) (0.56 mg/kg over 4 min).

Fifteen swine (*N* = 15, of which eight were from the baseline group) were subjected to a 90-min mid-LAD artery coronary occlusion followed by reperfusion. Swine were scanned post-AMI at 1–2 days (*N* = 9, of which 6 were from the baseline group), 1–2 weeks (*N* = 8, of which 4 swine were from day 1–2 group), or 4 weeks (*N* = 4, of which 2 were from week 1–2 group). Resting first-pass CMR and late gadolinium enhancement (LGE) were also performed post-AMI as a reference for MVO (note that stress response post-AMI was not part of the study design). To compare regional MBF measured post-AMI to that at baseline, cross-sectional analysis was used because every animal was not imaged at all four time points (baseline, day 1–2, week 1–2, and week 4).

### Data analysis

The left ventricular (LV) myocardium was manually segmented and divided into 6 segments following the American Heart Association (AHA) model [29] using a spatial-temporal averaging filter [30]. MBF was quantified using Buxton’s general kinetic model [31] described as follows:

$$ F=\frac{C-L}{2\cdotp B\cdotp {T}_D\cdotp \exp \left(-{T}_D/{T}_{1 blood}\right)}, $$

where F is measured MBF; C, L, and B refer to the mean myocardial signal in the control, labeled, and base image i.e. image acquired without the preceding labeling pulses; T_D_ is the post labeling delay, and T_1blood_ is the longitudinal relaxation time of blood, which was assumed to be 1650 ms [32].

Physiological noise (PN) is a measure of intra scan variability and is defined as the standard deviation of six repeated measurements of MBF in ml/g/min [18]. Segments with a temporal signal-to-noise ratio (tSNR = MBF/PN) < 2 in either rest or stress were excluded when analyzing regional MBF and vasodilator response in the baseline group. No data exclusion was applied in both baseline and post-AMI groups when comparing regional MBF measured post-AMI to that measured at baseline because the infarcted region (anteroseptal segment) is known in advance and expected to have low tSNR as a result of an AMI.

Based on LGE images, the anteroseptal segment was defined as the *infarcted* region. Three segments (inferior, inferolateral, and anterolateral) were considered to be the *remote* region. Resting MBF measured post-AMI from the infarcted and remote regions was compared against that measured at baseline.

The paired Student’s T-test was used to compare regional MBF at rest and stress in the baseline group. Comparison of regional MBF measured post-AMI to that at baseline was performed using an ordinary one-way ANOVA approach. The Holm-Sidak hypothesis test was used to correct for multiple comparisons that arose from serial sampling at different time points. A *P*-value < 0.05 was considered statistically significant. Values were reported as mean ± standard deviation (SD).

## Results

### MBF at rest

Sixty nine out of 246 (6 segments × 41 slices) segments were excluded from analysis due to low tSNR (tSNR< 2). Majority of excluded segments were from inferoseptal and inferior regions. The two segments account for approximately 70% of all excluded segments while each of the other four segments (i.e. inferolateral, anterolateral, anterior, and anteroseptal) only accounts for approximately 9% of all excluded segments.

Measured signal-to-noise-ratio (SNR) in the image without a labeling pulse was 98 ± 31 (range 37–155) and was similar to a previous study in humans where SNR was 90 ± 22 (range 53–110) [20]. At baseline (healthy state), regional MBF and PN were 1.08 ± 0.62 and 0.15 ± 0.10 (ml-blood/g-tissue/min), respectively.

### MBF at rest and stress

Segments with tSNR < 2 either at rest or stress were excluded from analysis. That results in 53 out of 150 segments were excluded from analysis. The mean ± standard deviation of heart rate across swine were 93 ± 9 and 87 ± 6 beats-per-minute at rest and stress, respectively.

**Fig. 2:**
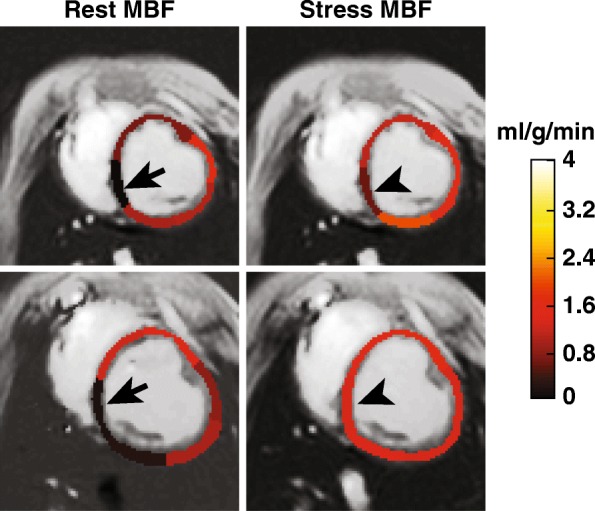
Rest and stress MBF maps from two representative healthy swine. Low MBF was observed in the inferior and inferoseptal segments at rest (arrows) but was elevated during vasodilation (arrow heads). Global MBF ± physiologic noise (PN) at rest and stress are (top row) 0.87 ± 0.04 and 1.38 ± 0.02 ml/g/min and (bottom row) 0.78 ± 0.16 and 1.39 ± 0.07 ml/g/min, respectively. Inferoseptal MBF ± PN at rest are (top) 0.03 ± 0.17 and (bottom) 0.13 ± 0.25 ml/g/min. These were elevated to 0.62 ± 0.09 and 1.31 ± 0.35 ml/g/min during vasodilation, respectively

**Fig. 3:**
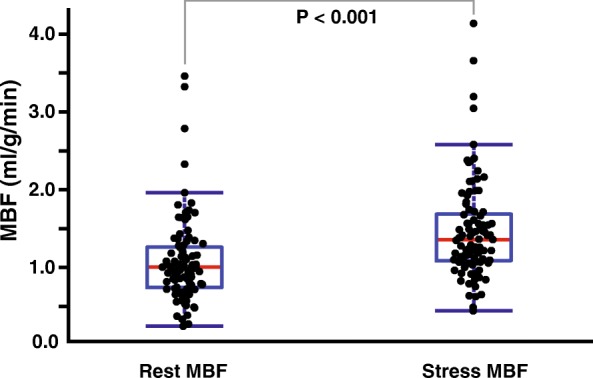
Box plot comparing regional rest and stress MBF measured from ASL-CMR. Regional MBF was significantly increased with vasodilation from 1.08 ± 0.54 to 1.47 ± 0.62 ml/g/min (*P* < 0.001). The central red line represents the median, the edges of the box are the 25th and 75th percentile, and the whiskers cover approximately 99.3% of all data

Regional MBF was significantly increased from 1.08 ± 0.54 to 1.47 ± 0.62 ml/g/min during dipyridamole vasodilator stress (*P* < 0.001). The regional myocardial perfusion reserve (MPR) was 1.51 ± 0.65, which corresponds to an MBF increase of 53% with vasodilation. MBF increase with dipyridamole vasodilator stress can be seen from representative MBF maps shown in Fig. 2. As seen in this figure, the inferoseptal segments have low MBF at rest (arrows) that become elevated with vasodilation (arrow heads). Regional MBF at rest and stress were compared against each other using box plot as seen in Fig. 3. The central red line represents the median, the edges of the box are the 25th and 75th percentile, and the whiskers extend to approximately 99.3% of all data.

### MBF at rest post-AMI

**Fig. 4:**
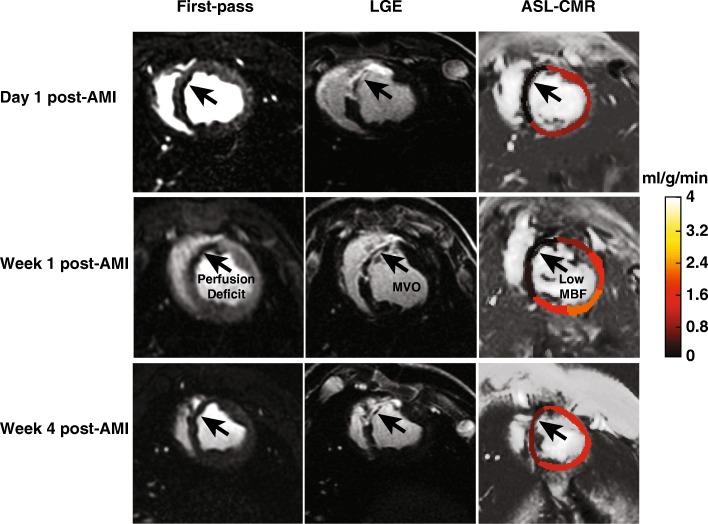
Representative resting MBF maps measured by ASL-CMR at 1 day, 1 week and 4 weeks post-AMI. Low MBF at rest measured in the infarcted region (arrows) is consistent with perfusion deficit seen on first-pass CMR and microvascular obstruction (MVO) seen on LGE

Significant reduction in MBF in the infarcted region compared to the remote region can be seen from the MBF maps acquired at 1 day, 1 week, and 4 weeks post-AMI (Fig. 4). As seen in this figure, low MBF measured by ASL-CMR in the infarcted region was consistent with perfusion deficit seen on first-pass CMR and MVO seen on LGE images (arrows).

**Fig. 5:**
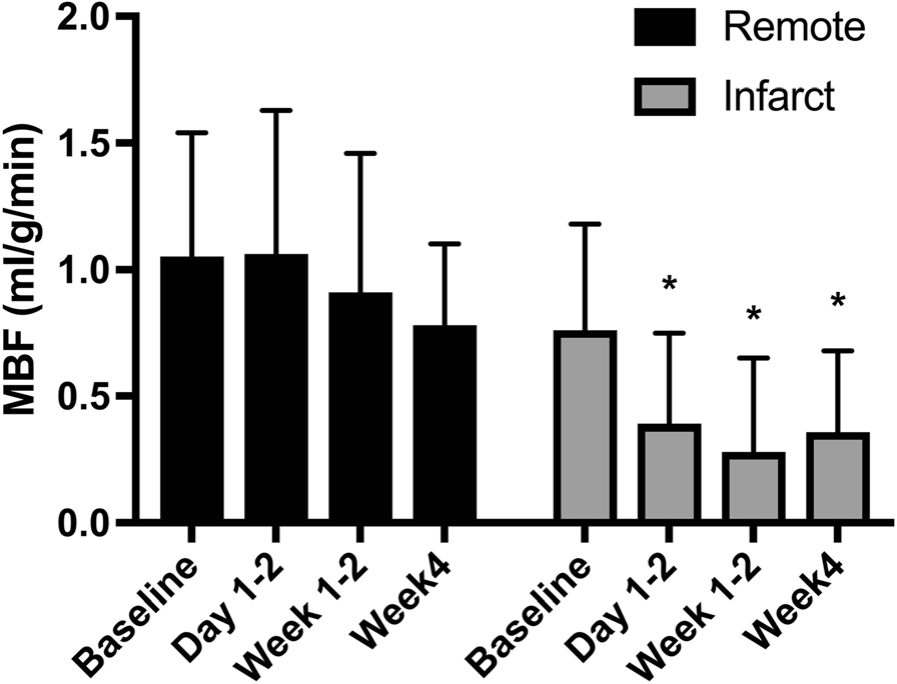
Regional resting MBF measured in remote (inferior, inferolateral, and anterolateral combined) and infarcted (anteroseptal) regions post-AMI and at baseline. Error bars represent group SD (standard deviation). In the infarcted region, significant reduction in MBF was seen in post-AMI groups compared to that at baseline (*P* < 0.03), as indicated by (*). There was no significant difference (*P* > 0.60) in measured MBF at all time points in the remote region

In the infarcted region, measured MBF post-AMI was significantly lower than that measured at baseline (*P* < 0.03) (Fig. 5). In the remote region, there was no significant difference in measured MBF post-AMI compared to that at baseline (*P* > 0.60).

## Discussion

This swine AMI study demonstrates that ASL-CMR can detect significant reduction in MBF in infarcted region consistent with perfusion deficit seen on first-pass CMR and MVO seen on LGE. ASL-CMR may potentially be used as an alternative to the gadolinium-based assessment of MVO with first-pass CMR and LGE. Additionally, ASL-CMR can quantify vasodilator response with dipyridamole infusion in healthy swine that may be useful for studying coronary and microvascular function. That is warranted in future study, in which ASL-CMR could be used to assess vasodilator response post-AMI.

At baseline, we found regional MBF using ASL-CMR was consistent with previous study that were 1.30 ± 0.60 and 1.00 ± 0.40 ml/g/min using first-pass CMR and microspheres, respectively [33]. Additionally, regional PN in this study (0.15 ± 0.10 ml/g/min) was comparable to a previous human study where measured PN was 0.21 ± 0.11 ml/g/min [20]. A post-labeling-delay of 1RR is used in human study, however, a 2RR post-labeling-delay was used in swine due to higher heart rate.

This study shows that ASL-CMR is able to detect vasodilator response in swine with dipyridamole infusion. MBF was increase from 1.08 ± 0.54 to 1.47 ± 0.62 ml/g/min (*P* < 0.001), which is consistent with a previous swine study [34], as listed in Table 2. That corresponds to approximately 53% increase in MBF with vasodilation. Quantitative assessment of vasodilator response plays an important role in studying microvascular dysfunction as seen in Uren et al., in which microvascular function was shown to be compromised in both the infarcted and the remote territories [7].

**Table 2:** Rest and stress myocardial blood flow (MBF) measured from ASL-CMR in comparison with literature values

	Technique	Rest MBF (ml/g/min)	Stress MBF (ml/g/min)
Schmitt et al., [33]	Microspheres	1.00 ± 0.40	NA
	First-pass CMR	1.30 ± 0.60	NA
Mahnken et al., [34]	CT perfusion	0.98 ± 0.19	1.34 ± 0.40
This Study	ASL-CMR	1.08 ± 0.54	1.47 ± 0.62

We observed a smaller vasodilator response (approximately 53%) compared to that of humans, where the MBF increase is approximately 300% [35]. It is possible that isoflurane anesthesia may cause the blunted vasodilator response as suggested by several previous studies [36–40]. Additionally, prolonged acquisition of the ASL-CMR sequence (3 min per slice) may result in faded vasodilator response since the effect of dipyridamole decays over time. Future studies may utilize invasive measurements of coronary pressure and flow to better monitor effects of anesthesia and vasodilation on coronary flow.

In a previous swine study, Poncelet et al. reported that MBF at rest was 1.50 ± 0.41 ml/g/min and increased by 150% to 3.76 ± 1.21 ml/g/min during peak hyperemia of 750 micro-gram/kg/min adenosine infusion [16]. It is noted that the dosage used in Poncelet’s study was more than five times the typical dose (140 micro-gram/kg/min) used in human. Both MBF and MPR reported by Poncelet et al., are higher than those in this study that we attribute to the differences in animal preparation, anesthesia, stress agent, and the dosage of the stress agent.

MVO is one of the most common complications after reperfusion [1] and is independently associated with adverse LV remodeling and poor patient prognosis [4–6, 41]. Therefore, early detection and serial assessment of MVO plays an important role in management of patient post-AMI that may improve patient prognosis and prevent recurrent AMI. At 1 day post-AMI, all swine demonstrated perfusion deficit seen on first-pass CMR and MVO seen on LGE within the infarcted territory. That is consistent with the previous studies [42–44], in which the 90-min mid-LAD occlusion model consistently creates a transmural infarction with MVO that is resolved by week 4 post-AMI. In this study, we have qualitatively evaluated the presence of MVO using first-pass CMR and LGE – these confirm that MVO is present at both day 1 and week 1 but is resolved by week 4. Therefore, it can be inferred that low MBF at the early time points is dominantly due to the presence MVO and that when it is resolved by week 4, low MBF still remains due to the absence of vessels in the infarcted region. Further studies with histological ground truth are needed to validate this hypothesis.

Serial assessment of regional MBF is potentially useful to monitor treatment efficacy, guide treatment plan, and develop of drugs and therapies. CT perfusion, SPECT, PET, and gadolinium-based first-pass CMR have been used for quantitative assessment of MBF. These imaging modalities may be limited for serial monitoring because they requires the use of ionizing radiation and/or exogenous contrast agents. ASL-CMR, on the other hand, is safe, repeatable, and a direct measure of tissue perfusion, that makes it a viable alternative.

### Limitations

There are several limitations in our study. Firstly, this is a cross-sectional study with a small sample size post-AMI, which hinders an interpretation of changes in the regional MBF over time because different animal groups may exhibit different infarct size and severity. Secondly, no gold standard method was used to validate the vasodilator response measured in the baseline group and the effect of anesthesia on coronary vasodilation was not monitored. Additionally, regional analysis was used in this study due to low SNR nature of ASL-CMR, therefore, it is not possible to differentiate MVO from the infarcted tissue.

A large number of segments were rejected at rest due to low tSNR. This is likely associated with coronary architecture, vascular resistance, regional variation in motion and filed inhomogeneity rather than sequence limitation. This is because the low tSNR issue does not occur in recent human studies using the same ASL-CMR sequence [20, 45, 46]. For example, only five out of 96 segments were excluded in Yoon et al., [46].

We did not observe any significant changes in MBF in remote myocardium post-AMI. This may be due to the low sample size at each time point post-AMI. Secondly, to observe the remote myocardial response, it is possible that the vasodilator response might need to be evaluated, which was not performed in this study. A previous study has demonstrated T2-BOLD response alterations in a porcine AMI model [47]; future studies could combine myocardial BOLD response and rest-stress ASL measurements.

## Conclusions

Non-gadolinium based ASL-CMR is able to quantitatively assess regional MBF at rest and under vasodilation in healthy swine, as well as detect changes in regional MBF post-AMI. ASL-CMR could potentially be used to detect and monitor microvascular injury/obstruction and microvascular function not only with infarcted myocardium, but also in salvageable and remote regions, which may be early indicators of downstream adverse remodeling processes post-injury.

## Abbreviations

AMI: Acute myocardial infarction
ASL: Arterial spin labeling
bSSFP: Balanced steady state free precession
CMR: Cardiovascular magnetic resonance
CT: Computed tomography
FAIR: Flow-sensitive alternating inversion recovery
LAD: Left anterior descending coronary artery
LGE: Late gadolinium enhancement
LV: Left ventricle/left ventricular
MBF: Myocardial blood flow
MPR: Myocardial perfusion reserve
MVO: Microvascular obstruction
PET: Positron emission tomography
PN: Physiological noise
RR: R-wave to R-wave duration
SNR: Signal-to-noise ratio
SPECT: Single photon emission computed tomography
tSNR: Temporal signal-to-noise ratio
